# Supplementary material for: Cross-ecosystem carbon flows connecting ecosystems worldwide
Source: Nat Commun. 2018 Nov 16;9:4825. doi: 10.1038/s41467-018-07238-2 (PMC6240079; doi:10.1038/s41467-018-07238-2)
Supplement: Supplementary file 1 — Supplementary Information [file 41467_2018_7238_MOESM1_ESM.pdf]

Cross-ecosystem carbon flows connecting ecosystems worldwide

Gounand et al.

Supplementary Information

Correspondence to: isabelle.gounand@eawag.ch

or florian.altermatt@eawag.ch

Content

Supplementary Figure 1 Ecosystem type - climatic zone combinations ………..…………… 2

Supplementary Table 1 Test of mean NEP difference from zero …..……………………..… 3

Supplementary Table 2 Definition of ecosystem and climate categories …………………… 4

Supplementary Table 3 Summary of cross-ecosystem spatial flow data …………………… 5

Supplementary Table 4 Summary of local ecosystem data …………………..…………… 6

Supplementary Table 5 Factors used for conversions into grams of carbon ……………...… 8

Supplementary Table 6 Sensitivity of spatial flows to the distance from shoreline ……….. 9

Supplementary Table 7 Definition of flow drivers ………………………………….………… 10

Supplementary References ……………………………………………………………….. 11

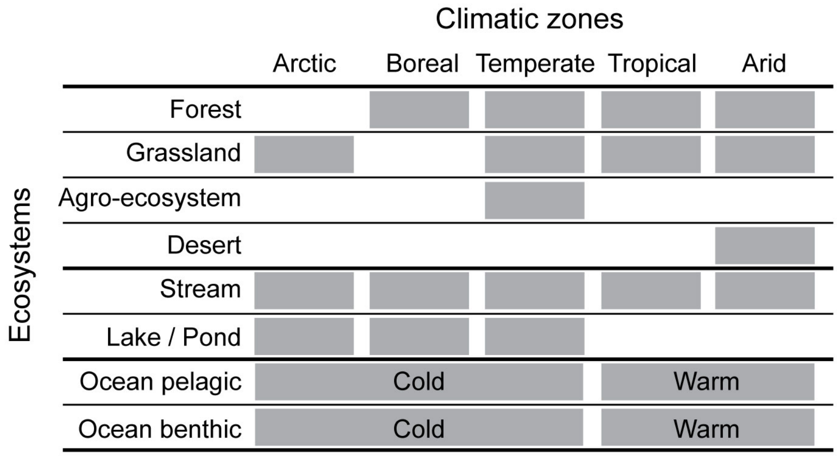

Supplementary Figure 1: Ecosystem type - climatic zone combinations considered in this study

We collected values of local carbon fluxes for all combinations marked by a grey rectangle, as well as cross-ecosystem spatial flows linking these ecosystems. The other combinations either do not exist, or do not have enough data available.

Supplementary Table 1: Test of the difference of mean Net Ecosystem Production from zero

Ecosystem type	t-statistic	df	p-value	mean	95% confidence interval
Forest	10.318	86	<0.001	252.32	[203.71; 300.94]
Grassland	2.5545	69	0.01285	125.86	[27.57; 224.16]
Agro-ecosystem	5.502	20	<0.001	317.56	[197.16; 437.96]
Desert	3.6173	14	0.002801	43.87	[17.86; 69.88]
Stream	-8.6537	128	<0.001	-249.59	[-306.66; -192.52]
Lake	-5.22552	88	<0.001	-23.14	[-31.89; -14.39]
Ocean pelagic	6.3366	70	<0.001	68.59	[47.00; 90.18]
Ocean benthic	1.0838	49	0.2838	70.45	[-60.17; 201.08]

Results of two-sided t-tests carried on the NEP data of each ecosystem type. Abbreviation “df” stands for degrees of freedom.

Supplementary Table 2: Definition of ecosystem and climate categories

	Definition	Example ecosystems
Climatic zones		
Arctic	Extreme temperature limitation of growing season length, with abiotic conditions not supporting tree growth in arctic, subarctic, and alpine zones; OR high latitude oceans, generally above 66.5°	Tundra (grassland)<br>Alpine grassland
Boreal	Strong temperature limitation of terrestrial growth, but environment supports tree growth. Covers northern parts of North America, Europe and Russia from latitudes 50° to 55°; OR oceans between 50° and 66.5°	Taiga (forest)<br>Sub-alpine forest
Temperate	Seasonal terrestrial growth with some temperature limitation. Covers latitudes between 23.5° and 50° to 55°, including oceans in this latitudinal range	Beech forest
Tropical	Warm terrestrial tropical, sub-tropical, equatorial systems not limited by drought between 0° to 23.5° latitude (including subtropical system), including oceans in this latitude range	Savanna (grassland), rainforest
Arid	Severely water-limited terrestrial systems at all latitudes, including arid, semi-arid, xeric, xerophytic, xeromorphic, Mediterranean systems, continental, warm or cold, and polar deserts	Garrigue (grassland), shrubland (grassland or forest, depending on the canopy), chaparral (grassland), steppe (grassland), caatinga (forest), cerrado
Ecosystems		
Forest	Complete vegetation cover with trees as dominant vegetation; tree canopy covers most of the surface	Rainforest, caatinga, woodland, some shrubland, cerrado
Grassland	Complete vegetation cover, but with only very few or no trees; vegetation dynamics dominated by water limitation, fires and grazing.	Steppe, savannah, meadow, prairie, tundra, old field, some shrublands, herbaceous rich-fen vegetation.
Desert	Extreme growth limitation by water availability, with little vegetation distributed in remote patches	Sandy land.
Agro-ecosystem	Ecosystems devoted to crop production or cattle grazing, often fertilized or irrigated to remove nutrient or water limitations for growth	Cropland, pasture.
Stream	Running freshwater and lotic systems of all sizes, including rivers	Creek, brook, river, stream
Lake	Standing (lentic) freshwater systems	Reservoir, lake, pond
Ocean	All salt water ecosystems with no emerged vegetation, including internal seas	Sea, ocean shelf, estuary, lagoon
Ocean pelagic	Ecosystems in the open water columns of oceans and seas	Upwelling system, open ocean
Ocean benthic	Ecosystems at the bottom of oceans and seas	Coral reef, sea grass bed, eelgrass meadow, kelp forest, deep-sea flour.

Supplementary Table 3: Summary of cross-ecosystem spatial flow data

Donor	Recipient	Material	n	Min	q25	Median	q75	Max	Data References
Forest	Agroeco.	Terr. plants	2	13.545	36.360	59.175	81.990	104.81	50
Forest	Stream	Terr. plants	96	0.2500	55.247	178.65	280.62	2085.8	8, 12, 13, 14, 15, 16, 17, 21, 26, 27, 28, 29, 30, 31, 33, 65, 78, 90, 96, 117, 118, 123, 124, 138, 203, 205, 212, 235, 375, 389, 392
Forest	Stream	Terr.inverteb.	49	0.1414	1.0683	3.0368	7.3321	20.386	2, 3, 4, 5, 6, 7, 23, 25, 32, 65, 98, 147, 365, 369, 370, 375, 525, 526
Forest	Lake	Terr. plants	9	1.2000	1.5439	6.4000	47.000	465.00	20, 109, 295, 316, 375, 520, 521
Forest	Lake	Terr.inverteb.	1	0.0184	0.0184	0.0184	0.0184	0.0184	375
Forest	Lake	Small amphib.	11	0.0331	0.4706	1.3528	3.7450	7.4100	60, 61
Forest	Lake	POC	2	1.8100	3.3259	4.8418	6.3576	7.8735	522
Forest	Ocean	Terr. plants	18	0.1000	27.249	153.80	312.00	492.80	430, 523
Forest	Ocean	Terr.inverteb.	5	0.1300	0.1700	0.8600	1.5100	1.6400	527
Forest	Ocean	POC	9	0.0003	9.1000	15.100	56.900	285.00	430
Forest	Ocean	DOC	7	0.1000	40.500	48.000	82.050	138.10	430
Grassland	Stream	Terr. plants	9	0.5500	16.000	64.593	250.00	265.77	16, 18, 65, 89, 117, 118, 138, 205
Grassland	Stream	Terr.inverteb.	6	0.1005	0.3413	0.8304	2.0334	2.5296	9, 32, 365
Grassland	Stream	Mammal faeces	2	502.00	746.75	991.50	1236.2	1481.0	364
Grassland	Stream	Carcass	1	136.80	136.80	136.80	136.80	136.80	556
Agroeco.	Forest	Mammal faeces	2	0.4829	0.6448	0.8067	0.9686	1.1305	367
Agroeco.	Stream	Terr. plants	23	1.4000	61.814	104.70	165.15	264.80	12, 78
Agroeco.	Stream	Terr.inverteb.	5	1.4544	4.4640	5.2699	6.1504	6.1504	3, 98, 365
Desert	Stream	Terr. plants	1	8.4000	8.4000	8.4000	8.4000	8.4000	94
Wetland	Forest	Small amphib.	1	64.634	64.634	64.634	64.634	64.634	59
Wetland	Grassland	Aqu. insects	6	0.0136	0.0339	0.1410	0.3297	2.4408	57, 66
Stream	Forest	Aqu. insects	30	0.0022	0.1377	0.4857	0.9384	8.3480	10, 11, 19, 65, 67, 74, 77, 78, 147, 266, 365, 366, 477
Stream	Forest	Carcass	14	0.0693	1.7076	2.9078	5.8411	15.980	386, 390
Stream	Grassland	Aqu. insects	5	0.0027	0.0628	0.1329	0.2828	0.9516	65, 77, 365
Stream	Agroeco.	Aqu. insects	9	0.0067	0.2648	1.4607	4.3822	9.6100	69, 77, 78, 365
Stream	Desert	Aqu. insects	4	0.1742	1.3000	1.8098	2.7536	5.1815	22, 34, 387
Lake	Forest	Aqu. insects	14	0.0976	0.7246	1.8110	9.1252	467.87	62, 368, 374, 518, 524, 528
Lake	Forest	Small amphib.	11	0.0030	0.0273	0.0914	41.002	127.33	58, 60, 528
Lake	Grassland	Aqu. insects	20	0.3057	1.2527	4.7484	24.641	467.87	1, 24, 62, 368, 374, 383, 517, 519
Lake	Desert	POC	1	0.1530	0.1530	0.1530	0.1530	0.1530	56
L. Pelagic	L. Benthic	Phytoplankton	3	1.0971	8.1016	15.106	19.965	24.824	99, 134, 379
L. Pelagic	L. Benthic	Zooplankton	1	24.983	24.983	24.983	24.983	24.983	379
L. Pelagic	L. Benthic	POC	13	2.1448	13.950	20.925	32.860	141.82	294, 296, 485
Ocean	Forest	POC	1	0.1000	0.1000	0.1000	0.1000	0.1000	430
Ocean	Forest	DOC	6	0.4000	8.7500	15.900	20.350	67.300	430
Ocean	Desert	Macroalgae	6	44.520	93.921	835.79	1638.3	2646.9	103, 104, 371
Ocean	Desert	Carcass	3	4.9500	10.125	15.300	19.575	23.850	104
Ocean	Stream	Carcass	3	59.667	61.075	62.483	66.847	71.211	389
Oc. Pelagic	Oc. Benthic	Macroalgae	1	0.1786	0.1786	0.1786	0.1786	0.1786	503
Oc. Pelagic	Oc. Benthic	Phytoplankton	16	0.0362	0.1222	0.4978	2.7648	142.00	310, 311, 499, 539
Oc. Pelagic	Oc. Benthic	Zooplankton	38	0.3167	0.7563	1.5629	6.4680	156.90	308, 311, 384, 385, 495, 539, 557
Oc. Pelagic	Oc. Benthic	Carcass	4	0.0157	0.1134	0.1556	0.5290	1.6200	499, 500, 503, 504
Oc. Pelagic	Oc. Benthic	POC	40	0.1330	5.7875	19.444	70.250	562.91	80, 102, 116, 131, 133, 304, 306, 311, 319, 330, 334, 335, 338, 393, 494, 514
Oc. Benthic	Desert	Macroalgae	7	0.4795	176.46	1522.1	4730.8	17,365	372, 373, 382
Oc. Benthic	Oc. Benthic	Macroalgae	3	115.80	124.65	133.50	148.75	164.00	418, 420

Agroeco. = Agro-ecosystem; Oc. = Ocean; Terr. = Terrestrial; Aqu. = Aquatic; inverteb. = invertebrates; amphib. = amphibians; POC = Particulate Organic Carbon; DOC = Dissolved Organic Carbon. We provide the number of data points (n), the minimum, 25% quartile (q25), median, 75% quartile (q75) and maximum values of estimates distributions for each pair of ecosystems coupled and per type of the material exchanged, as well as the associated references from which we extracted the data, listed in Supplementary Data 1. Values are expressed in gC m^{-2} $year^{-1}$. Donor and Recipient refer respectively to the ecosystem from which comes and to which go the spatial flow.

Supplementary Table 4: Summary of local ecosystem data

Ecosystem	Variable	n	Min	q25	Median	q75	Max	Data References
Forest	GPP	74	387.00	1064.0	1379.5	1786.0	4188.0	38, 39, 41, 42, 49, 52, 145, 146, 155, 156, 157, 158, 160, 161, 162, 163, 167, 169, 170, 171, 174, 250, 257, 283, 288, 289, 545
Forest	R	75	131.00	825.00	1098.0	1333.2	3860.0	38, 39, 41, 42, 49, 52, 108, 145, 146, 155, 156, 157, 158, 160, 161, 162, 163, 170, 171, 174, 249, 250, 257, 283, 288, 289, 545
Forest	NEP	87	-146.00	101.50	200.00	336.00	976.00	38, 39, 41, 42, 49, 145, 146, 151, 155, 156, 157, 158, 160, 161, 162, 163, 165, 166, 168, 170, 171, 174, 249, 250, 257, 263, 271, 280, 283, 287, 288, 289, 398, 421, 545
Forest	Detritus	90	70.871	447.88	1040.0	2491.9	24,980	36, 37, 38, 40, 41, 42, 63, 64, 73, 148, 149, 150, 151, 153, 154, 164, 173, 183, 224, 226, 248, 258, 259, 352, 398, 422, 529, 530
Forest	D. rate	78	0.0120	0.2371	0.4334	0.9717	66.000	91, 95, 122, 135, 152, 176, 184, 201, 224, 225, 226, 228, 229, 248, 249, 251, 252, 253, 255, 256, 354, 356, 358
Grassland	GPP	77	36.500	232.00	611.53	1047.4	3723.0	47, 54, 81, 86, 87, 88, 92, 100, 167, 177, 178, 188, 189, 190, 257, 278, 279, 283, 285, 286, 394, 400, 403, 404, 405, 408, 410, 411, 412, 427, 439, 452
Grassland	R	62	52.900	235.50	556.38	1023.0	5725.6	35, 47, 54, 70, 71, 72, 76, 81, 86, 87, 88, 92, 100, 177, 178, 189, 257, 260, 278, 279, 283, 285, 286, 394, 400, 405, 408, 409, 410, 411, 412, 427, 439, 452, 531
Grassland	NEP	70	-212.00	-34.750	52.884	119.92	2810.5	47, 54, 81, 86, 87, 88, 92, 100, 168, 172, 177, 178, 189, 257, 260, 263, 278, 279, 280, 282, 283, 285, 286, 377, 394, 397, 400, 405, 408, 410, 411, 412, 427, 439, 452
Grassland	Detritus	64	3.7568	88.198	176.81	279.35	2250.0	44, 46, 51, 54, 75, 85, 150, 151, 175, 180, 187, 282, 377, 378, 399, 406, 410, 413, 414, 415, 424, 426, 429, 432, 435, 436, 437, 530, 531
Grassland	D. flux	5	10.585	12.447	13.104	13.322	20.988	376
Grassland	D. rate	64	0.0120	0.1863	0.6230	1.0227	3.3731	91, 119, 120, 121, 125, 175, 179, 181, 182, 185, 186, 187, 194, 195, 227, 236, 284, 376, 407, 413, 415, 423, 425, 426, 531
Agroeco.	GPP	24	599.00	1286.8	1772.4	1992.6	2520.7	167, 232, 257, 277, 279, 281, 402, 428
Agroeco.	R	22	714.00	1066.9	1309.5	1563.1	1996.0	76, 257, 277, 279, 281, 402, 428
Agroeco.	NEP	21	-115.00	155.00	303.00	361.05	1204.5	168, 257, 277, 279, 281, 402, 428
Agroeco.	Detritus	14	19.520	134.53	153.75	213.30	379.22	381, 429, 431, 438, 453
Agroeco.	D. flux	3	68.750	69.875	71.000	99.000	127.00	106
Agroeco.	D. rate	12	0.5209	0.8503	1.1250	1.6598	2.6593	273, 380, 381, 416, 453, 454
Desert	GPP	12	20.000	76.598	106.44	138.49	220.00	396, 402, 440, 441, 442, 443, 445, 446
Desert	R	12	33.797	52.865	92.442	133.57	180.00	396, 402, 441, 442, 445, 446, 449, 450
Desert	NEP	15	-30.000	7.7626	32.596	62.483	130.41	151, 280, 391, 395, 396, 402, 441, 442, 444, 445, 446
Desert	Detritus	11	0.0000	0.9022	13.050	125.83	217.35	151, 406, 434, 447, 448, 450
Desert	D. rate	12	0.0250	0.2672	0.5275	0.7105	1.4268	91, 237, 254, 433, 451
Stream	GPP	128	0.0000	13.470	55.240	155.36	1770.0	12, 15, 18, 28, 89, 90, 93, 94, 96, 97, 110, 111, 138, 159, 203, 207, 209, 210, 214, 218, 234, 235, 239, 261, 264, 266, 267, 268, 270, 275, 388, 389, 401, 455, 456, 465, 467, 547, 548
Stream	2nd Prod.	107	0.1132	2.1238	5.5056	14.175	287.75	13, 22, 93, 117, 215, 238, 239, 240, 241, 242, 243, 244, 245, 246, 265, 266, 368, 387, 388, 458, 459, 460, 472, 476, 477
Stream	R	136	2.0323	76.093	290.41	617.58	2148.9	12, 15, 28, 89, 93, 96, 110, 111, 138, 159, 203, 207, 208, 210, 214, 218, 234, 235, 245, 261, 264, 266, 267, 268, 270, 275, 401, 455, 456, 464, 465, 467, 546, 547, 548, 549
Stream	NEP	129	-1,795.3	-400.46	-114.10	-20.900	568.95	12, 15, 28, 33, 89, 93, 96, 110, 111, 138, 159, 203, 207, 209, 210, 214, 218, 234, 235, 261, 262, 264, 267, 268, 270, 271, 274, 275, 276, 401, 455, 456, 465, 467, 547, 548
Stream	Detritus	139	0.0941	11.376	56.500	281.67	6475.9	10, 13, 14, 28, 33, 67, 89, 90, 93, 94, 96, 124, 202, 204, 206, 210, 211, 212, 213, 217, 235, 238, 239, 240, 242, 244, 266, 321, 388, 392, 417, 457, 466, 470, 471, 474, 475, 540, 541, 542, 543, 544, 549
Stream	D. rate	100	0.1508	1.9109	4.0954	9.5022	203.90	8, 136, 137, 143, 144, 191, 192, 193, 196, 197, 198, 199, 200, 201, 216, 219, 230, 269, 353, 354, 355, 357, 359, 360, 361, 362, 363, 366, 389, 392, 468, 469
Lake	GPP	42	0.5270	16.796	53.050	161.67	1922.0	20, 53, 79, 101, 112, 114, 115, 290, 292, 293, 295, 297, 316, 317, 318, 323, 336, 379, 487, 488, 489, 490, 532, 535, 536
Lake	2nd Prod.	66	0.0200	1.6521	6.1314	19.443	442.00	246, 247, 290, 291, 295, 332, 336, 368, 473, 482
Lake	R	44	10.092	17.653	37.999	85.757	1100.5	53, 101, 113, 115, 290, 292, 293, 295, 297, 298, 336, 487, 488, 489, 490, 532, 533, 534, 535, 536
Lake	NEP	89	-230.02	-27.512	-12.865	-4.9348	65.922	53, 101, 109, 115, 271, 272, 274, 276, 290, 292, 293, 295, 297, 336, 462, 487, 488, 489, 490, 532, 535, 536
Lake	Detritus	43	0.7500	2.7875	4.7693	33.667	3840.0	53, 68, 298, 307, 320, 322, 323, 336, 379, 461, 463, 485, 486, 491, 537, 538
Lake	D. flux	1	49.268	49.268	49.268	49.268	49.268	485
Lake	D. rate	54	0.0167	0.2331	0.9424	1.9288	92.646	68, 105, 107, 139, 140, 141, 142, 231, 272, 316, 323, 376, 379, 478, 479, 480, 481, 483, 484, 486, 492, 493
Oc. Pelagic	GPP	66	0.7100	66.025	114.50	242.49	748.13	55, 80, 102, 116, 126, 128, 129, 130, 131, 132, 133, 304, 305, 309, 310, 311, 312, 313, 314, 315, 319, 329, 335, 337, 339, 341, 349, 351, 494, 516
Oc. Pelagic	2nd Prod.	22	2.4360	7.1014	13.277	23.067	122.63	319, 341, 351, 460, 494, 502, 503, 508, 550, 551, 552, 553, 554, 555
Oc. Pelagic	R	24	4.7433	55.635	112.36	217.95	521.28	304, 305, 313, 314, 315, 319, 329, 335, 337, 339, 341, 344, 349, 351, 494, 516
Oc. Pelagic	NEP	71	-69.927	17.507	40.000	86.868	447.00	43, 45, 48, 116, 304, 305, 313, 314, 315, 319, 329, 330, 337, 339, 341, 348, 349, 351, 494, 507, 516
Oc. Pelagic	Detritus	36	0.0180	2.0595	4.5426	12.660	334.49	129, 130, 311, 315, 319, 328, 334, 337, 338, 339, 344, 348, 349, 351, 457, 494, 513, 514, 516

Oc. Pelagic	D. rate	31	0.6492	2.1721	9.8550	44.632	788.40	126, 127, 132, 133, 319, 327, 328, 376, 495, 515
Oc. Benthic	GPP	54	10.000	83.365	240.71	745.20	4369.1	82, 83, 84, 221, 222, 223, 299, 300, 301, 311, 313, 315, 324, 333, 339, 340, 343, 344, 349, 350, 506
Oc. Benthic	2nd Prod.	42	0.3519	6.7409	11.400	25.715	745.90	311, 331, 350, 494, 501, 502, 503, 504, 506, 507, 508, 510, 511, 512
Oc. Benthic	R	47	2.4717	84.390	253.43	570.15	2737.5	83, 221, 222, 223, 233, 299, 300, 301, 304, 305, 311, 313, 315, 319, 324, 331, 333, 339, 340, 344, 349, 350
Oc. Benthic	NEP	50	-401.50	-103.70	-4.1724	39.542	2120.7	83, 116, 220, 221, 222, 223, 299, 300, 301, 311, 313, 315, 324, 331, 333, 339, 340, 344, 348, 349, 350, 420, 507
Oc. Benthic	Detritus	51	0.0250	8.5950	324.01	976.10	3520.0	300, 302, 303, 311, 319, 325, 326, 339, 347, 348, 350, 498, 501, 502, 503, 504, 505, 507, 508, 509
Oc. Benthic	D. flux	2	15.600	47.700	79.800	111.90	144.00	342, 393
Oc. Benthic	D. rate	38	0.3855	1.2632	3.4537	6.9958	32.589	303, 326, 345, 346, 347, 376, 418, 419, 420, 496, 497, 499

Oc. = Ocean; GPP = Gross Primary Production; R = ecosystem respiration; NEP = Net Ecosystem Production; 2nd Prod. = Secondary production; D. = Decomposition. We provide the number of data points (n), the minimum, first quartile (q25), median, third quartile (q75) and maximum values of estimate distributions for each ecosystem x variable combination, as well as the associated references from which we extracted the data, listed in Supplementary Data 1. Values of stocks (Detritus), fluxes (GPP, R, NEP, 2nd Prod, and D. flux) and rates (D. rates) are expressed in gC m^{-2}, gC m^{-2} $year^{-1}$ and $year^{-1}$, respectively.

Supplementary Table 5: Factors used for conversions into grams of carbon

Type of material	KJ	Kcal	molC	gCO_2	gO_2	$molO_2$	g WW	g DW	g AFDW
Organic Tissue [a]	0.02 (0.07%)	0.09 (0.10%)	12 (2.11%)	0.2727 (3.92%)			0.09 (1.48%)	0.45 (4.09%)	0.5 (7.02%)
Productivity, photosynthetic quotient = 1.2 [b]					0.3125 (3.03%)	10 (0,26%)			
Respiration, respiration quotient = 1[ab]					0.375 (3.72%)	12 (0.30%)			
Non woody primary producer terrestrial [c]								1/3.33* (2.04%)	
Algae, sea grasses [c]							1/16.7 (0.10%)	1/2.92 (0.89%)	
Salamanders[d]								0.473 (0.33%)	
Arthropods[e]								0.496 (4.25%)	

KJ = kilojoule; Kcal = kilocalorie; molC = mole of carbon; gCO_2 = gram of carbon dioxide; gO_2 = gram of di-oxygen; $molO_2$ = mole of di-oxygen; gWW = gram of wet weight; dDW = gram of dry weight; gAFDW = gram of ash-free dry weight. Values into brackets give the percentage of raw values converted using a given factor. 50.16% of the values were already provided in carbon units.

[a] from Table 1 p26 of supplementary reference[1], and references therein.
[b] from supplementary references[2–4].
[c] from Table 2.5 p26 of supplementary reference[5].
[d] from supplementary reference[6].
[e] from supplementary reference[7].
* used general conversion factor 0.45 in desert and arid grassland where the vegetation comprise woody species.

Supplementary Table 6: Sensitivity of spatial flows to the distance from shoreline

Recipient Ecosystem	Flow origin	10m from shoreline	100m from shoreline	% of affected data points
Forest	Invertebrates	**0.71**	**0.08**	82
Forest	Vertebrates	**2.12**	**0.29**	93
Forest	POC-DOC	13.1	13.1	0
Grassland	Invertebrates	**1.34**	**0.18**	74
Agro-ecosystem	Primary producers	59.18	59.18	0
Agro-ecosystem	Invertebrates	**1.46**	**1.46**	44
Desert	Primary producers	**1522.07**	**152.21**	100
Desert	Invertebrates	**1.81**	**0.18**	100
Desert	Vertebrates	**15.3**	**1.53**	100
Desert	POC-DOC	0.15	0.15	0
Stream	Primary producers	153.6	153.6	0
Stream	Invertebrates	2.78	2.78	0
Stream	Vertebrates	104.01	104.01	0
Lake	Primary producers	6.8	6.8	0
Lake	Invertebrates	12.5	12.5	0
Lake	Vertebrates	1.35	1.35	0
Lake	POC-DOC	18.6	18.6	0
Ocean Benthic	Primary producers	3.8	3.8	0
Ocean Benthic	Invertebrates	1.51	1.51	0
Ocean Benthic	Vertebrates	0.16	0.16	0
Ocean Benthic	POC-DOC	25.46	25.46	0

Values in columns 3 and 4 are the medians of the spatial inflows of different origins entering recipient different ecosystem types in gC m^{-2} $year^{-1}$. In the third column we assume that lateral flows are deposited within 10 meters from the shoreline (as in Figure 3). In the fourth column we assume they are deposited within 100 m. Shading and bold values highlight cases where flows are affected by this assumption.

Supplementary Table 7: Definition of flow drivers

	Definition	Example cross-ecosystem spatial flow
Passive drivers		*With reference to studies included in this study*
Fall / Wind	Movement of terrestrial particles, detritus, or organisms simply falling or windblown	Tree leaves windblown in lakes[53]; terrestrial arthropods falling in streams[3]; mangrove arboricole crabs falling in water and eaten by fish[527]
Leaching	Movement of small particles or dissolved molecules through diffusion in aquatic medium	Dissolved or particle organic carbon diffusing[430]
Current / Tide	Lateral movement of particles, detritus or organisms triggered by water currents or tides	Wrack grounding on beech[372]; macroalgal drift[420]; carcass deposited on beach[104]
Sinking	Vertical movements particles, detritus or organisms triggered by gravity along the water column	"Marine snow"[80], which can comprise phytoplankton sinking[99], zooplankton faecal pellets[308], salp carcasses[495]; fishery discard[503]
Active drivers		
Foraging	Daily movements of organisms to find food which trigger cross-ecosystem movement of resources (only those are considered here)	Deer grazing in agro-ecosystems and defecating in forests[367]; hippopotamus grazing in savannah and defecating in river[364]; zooplankton eating in pelagic area and finding refuge in deep sea[384]; bears bringing salmon carcasses on land[386].
Migration	Seasonal long-distance movement of populations triggering cross-ecosystem movement of resources	Downing of wildebeest during seasonal migration[556].
Life-cycle	Cross-ecosystem organismal movements required to complete a life-cycle	Emergence of aquatic insects to land[1]; salamander egg deposition in or emergence from aquatic systems[60]; anadromous salmon dying after breeding in freshwater systems[389].

References are provided in Supplementary Data 1.

Supplementary references

1. Weathers, K. C., Strayer, D. L. & Likens, G. E. in 25–26 (Academic Press, 2013). doi:[https://doi.org/10.1016/B978-0-08-091680-4.00024-X]
2. Duarte, C. M. *et al.* Seagrass community metabolism: Assessing the carbon sink capacity of seagrass meadows. *Global Biogeochem. Cycles* **24,** 1–8 (2010).
3. Irons III, J. G. & Oswood, M. W. Organic matter dynamics in 3 subarctic streams of interior Alaska, USA. *J N Am Bethol Soc* **16,** 23–27 (1997).
4. Huchette, S. M. H., Beveridge, M. C. M., Baird, D. J. & Ireland, M. The impacts of grazing by tilapias (Oreochromis niloticus L.) on periphyton communities growing on artificial substrate in cages. *Aquaculture* **186,** 45–60 (2000).
5. Opitz, S. *Trophic interactions in Caribbean coral reefs*. (International Center for Living Aquatic Resources Management, 1996).
6. Semlitsch, R. D., O'Donnell, K. M. & Thompson III, F. R. Abundance, biomass production, nutrient content, and the possible role of terrestrial salamanders in Missouri Ozark forest ecosystems. *Can. J. Zool.* **92,** 997–1004 (2014).
7. Small, G. E., Torres, P. J., Schweizer, L. M., Duff, J. H. & Pringle, C. M. Importance of terrestrial arthropods as subsidies in lowland Neotropical rain forest stream ecosystems. *Biotropica* **45(1),** 80–87 (2013).
