# Supplementary material for: Cross-ecosystem carbon flows connecting ecosystems worldwide
Source: Nat Commun. 2018 Nov 16;9:4825. doi: 10.1038/s41467-018-07238-2 (PMC6240079; doi:10.1038/s41467-018-07238-2)
Supplement: Supplementary file 3 — Description of Additional Supplementary Files [file 41467_2018_7238_MOESM3_ESM.pdf]

Description of Additional Supplementary Files

File Name: Supplementary Data 1
Description: References from which the data are extracted
