# Supplementary material for: Cross-ecosystem carbon flows connecting ecosystems worldwide
Source: Nat Commun. 2018 Nov 16;9:4825. doi: 10.1038/s41467-018-07238-2 (PMC6240079; doi:10.1038/s41467-018-07238-2)
Supplement: Supplementary file 5 — Reporting Summary [file 41467_2018_7238_MOESM5_ESM.pdf]

nature research

Corresponding author(s): Isabelle Gounand and Florian Altermatt

Reporting Summary

Nature Research wishes to improve the reproducibility of the work that we publish. This form provides structure for consistency and transparency in reporting. For further information on Nature Research policies, see Authors & Referees and the Editorial Policy Checklist.

Statistical parameters

When statistical analyses are reported, confirm that the following items are present in the relevant location (e.g. figure legend, table legend, main text, or Methods section).

n/a	Confirmed	
☐	☒	The exact sample size (n) for each experimental group/condition, given as a discrete number and unit of measurement
☐	☒	An indication of whether measurements were taken from distinct samples or whether the same sample was measured repeatedly
☐	☒	The statistical test(s) used AND whether they are one- or two-sided<br>*Only common tests should be described solely by name; describe more complex techniques in the Methods section.*
☒	☐	A description of all covariates tested
☐	☒	A description of any assumptions or corrections, such as tests of normality and adjustment for multiple comparisons
☐	☒	A full description of the statistics including central tendency (e.g. means) or other basic estimates (e.g. regression coefficient) AND variation (e.g. standard deviation) or associated estimates of uncertainty (e.g. confidence intervals)
☐	☒	For null hypothesis testing, the test statistic (e.g. F, t, r) with confidence intervals, effect sizes, degrees of freedom and P value noted<br>*Give P values as exact values whenever suitable.*
☒	☐	For Bayesian analysis, information on the choice of priors and Markov chain Monte Carlo settings
☒	☐	For hierarchical and complex designs, identification of the appropriate level for tests and full reporting of outcomes
☒	☐	Estimates of effect sizes (e.g. Cohen's d, Pearson's r), indicating how they were calculated
☐	☒	Clearly defined error bars<br>*State explicitly what error bars represent (e.g. SD, SE, CI)*

Our web collection on statistics for biologists may be useful.

Software and code

Policy information about availability of computer code

Data collection	Data collection based on literature review (full list of literature/references used is given). No software used.
Data analysis	For the analysis, we used the open source software R version 3.3.3, and the R-packages ggmap (version 2.6.1), maps (versin 3.2.0) and pgirmess (version 1.6.6)

For manuscripts utilizing custom algorithms or software that are central to the research but not yet described in published literature, software must be made available to editors/reviewers upon request. We strongly encourage code deposition in a community repository (e.g. GitHub). See the Nature Research guidelines for submitting code & software for further information.

Data

Policy information about availability of data

All manuscripts must include a data availability statement. This statement should provide the following information, where applicable:

- Accession codes, unique identifiers, or web links for publicly available datasets
- A list of figures that have associated raw data
- A description of any restrictions on data availability

Data that support the findings of this study are summarized in Supplementary Table 3 and Supplementary Table 4, with all associated references in Supplementary Data 1. Source data files are available from the corresponding authors upon request.

Field-specific reporting

Please select the best fit for your research. If you are not sure, read the appropriate sections before making your selection.

☐ Life sciences ☐ Behavioural & social sciences ☒ Ecological, evolutionary & environmental sciences

For a reference copy of the document with all sections, see nature.com/authors/policies/ReportingSummary-flat.pdf

Ecological, evolutionary & environmental sciences study design

All studies must disclose on these points even when the disclosure is negative.

Study description	Synthesis of data, collected from the literature, quantifying within and between ecosystem fluxes.
Research sample	Measurements of ecosystem fluxes: Gross Primary Production, Ecosystem Respiration, Secondary Production, Decomposition, flow between ecosystems, which could consist of different material types (e.g., detritus, insects, dissolved organic carbon).
Sampling strategy	No collection of data in the field (literature review; full reference list of literature used given).
Data collection	No collection of data in the field (literature review; full reference list of literature used given).
Timing and spatial scale	No collection of data in the field (literature review; full reference list of literature used given).
Data exclusions	No data were excluded from the analysis (statistical analysis on Net Ecosystem Production).
Reproducibility	Literature review; full reference list of literature on data used in the figures given.
Randomization	Not applicable: this is not an experimental study.
Blinding	Not applicable: this is not an experimental study.

Did the study involve field work? ☐ Yes ☒ No

Reporting for specific materials, systems and methods

Materials & experimental systems

n/a	Involved in the study
☒	☐ Unique biological materials
☒	☐ Antibodies
☒	☐ Eukaryotic cell lines
☒	☐ Palaeontology
☒	☐ Animals and other organisms
☒	☐ Human research participants

Methods

n/a	Involved in the study
☒	☐ ChIP-seq
☒	☐ Flow cytometry
☒	☐ MRI-based neuroimaging
